# Bipolar disorder in children and adolescents: diagnostic inpatient rates from 2000 to 2013 in Germany

**DOI:** 10.1186/s40345-016-0064-2

**Published:** 2016-11-11

**Authors:** Pradeep Rao, Julie K. Moore, Richard Stewart, Kevin Runions, Natasha Bear, Janice W. Y. Wong, Martin Holtmann, Florian D. Zepf

**Affiliations:** 1 Centre & Discipline of Child and Adolescent Psychiatry, Psychosomatics and Psychotherapy; School of Psychiatry and Clinical Neurosciences & School of Paediatrics and Child Health; Faculty of Medicine, Dentistry and Health Sciences, The University of Western Australia, Perth, Australia; 2 Department of Health, Western Australia, Community Child and Adolescent Mental Health Services, Perth, WA Australia; 3 Paediatric Consultation-Liaison Program, Department of Health, Western Australia, Child and Adolescent Mental Health Services, Perth, WA Australia; 4 Telethon Kids Institute, The University of Western Australia, Perth, Australia; 5 Department of Clinical Research and Education, Child and Adolescent Health Service, Department of Health, Western Australia, Perth, WA Australia; 6 Hospital for Child and Adolescent Psychiatry, LWL University Hospital of the Ruhr University Bochum, Hamm, Germany; 7 Department of Health in Western Australia, Specialised Child and Adolescent Mental Health Services (CAMHS), Perth, Australia

**Keywords:** Bipolar disorder, Children, Adolescents, Discharge diagnostic rates, Inpatients, Germany

## Abstract

**Background:**

Despite growing consensus on nosology and epidemiology of bipolar disorder (BD) in minors, differences remain. We contribute to this discussion by measuring long-term trends in the inpatient discharge rates of BD in minors.

**Methods:**

Nationwide German inpatient discharge diagnoses of BD and other related psychiatric disorders were mapped between 2008 and 2013 using registry data from the German Federal Health Monitoring System. This was compared with previously published data, 2000–2007, to assess long-term trends in diagnosis of BD at discharge. Long-term trends (2000–2013) were also computed.

**Results:**

Discharge diagnosis of BD increased by 18% (2.02–2.46 per 100,000) in minors. There was a significant increase of 24.1% in adolescents 15–19 years old (6.56–8.14 per 100,000). BD, at discharge, as a proportion of all psychiatric disorders, increased from 0.26% in 2008 to 0.27% in 2013. When analysing long-term trends (2000–2013), the rates for BD increased significantly as did trends for all mental disorders, except for psychotic disorders, which fell by almost 14%. Between 2000 and 2013, the rate for depression in minors increased by 730%.

**Limitations:**

The dataset consisted of cross-sectional administrative data points with diagnoses based on clinical criteria.

**Conclusions:**

The rate of BD as a discharge diagnosis in German minors has increased significantly, consistently exceeding the general trend for a rise in rates for mental disorders. Overall, the rate of discharge diagnosis of BD from inpatient units in Germany remains a small proportion of all psychiatric diagnoses.

## Background

Health policy and planning to address child mental health service delivery is dependent on clear data indicating the prevalence and hospitalisation rates for specific psychiatric disorders. In 2010, Holtmann et al. described the national trend rates of bipolar disorder (BD) in Germany between 2000 and 2007. They found an increase in rate of diagnosis at discharge of BD in German children and adolescents that exceeded the general trend for increased mental disorder diagnoses. When compared to data from the USA (Harpaz-Rotem and Rosenheck 2004; Moreno et al. 2007), the authors concluded that a diagnosis of BD in Germany was comparatively rare. Hypothesised reasons for the difference in prevalence included a possible reluctance to use BD as a diagnostic category for young patients in Germany, differences in diagnostic criteria between ICD-10 (World Health Organization 1992) and DSM-IV TR (American Psychological Association 1994) criteria, a higher frequency of stimulant and antidepressant use in the US, transatlantic differences in aggressive pharmaceutical marketing strategies as well as potential ‘diagnostic up-coding’ for reimbursement for diagnostic reasons.

The low prevalence of paediatric bipolar disorder (PBD) diagnoses also appears to be the case in other European countries. For instance, a survey conducted by the Office of National Statistics (Green 2005) on common childhood mental disorders in the UK did not mention BD, mania or hypomania throughout the document. A review on an international perspective on BD in children and adolescents (Soutullo et al. 2005) stated that considerable scepticism existed in Europe over the high prevalence of PBD in the US. The authors concluded that PBD was ‘fairly rare’ outside the US in epidemiological samples as well as inpatient samples. The authors hypothesised a relative lack of data, differences in diagnostic criteria, clinician bias towards other diagnoses such as hyperkinetic, conduct, depressive and personality disorders, possible over diagnosis in the US and/or a true higher prevalence of paediatric BD in the US as possible explanations. If rates would reflect a true prevalence, it could be possible that Europe has lagged behind the US in a secular trend. A recent meta-analysis (Van Meter et al. 2011) suggests there is much less variation in international prevalence but the range of variation in the United States is shown to be high (0.1–6.7%) Notably, this meta-analysis included bipolar disorder subtypes and data from outpatient settings.

The age of onset of BD also significantly differs, with one study (Post et al. 2008) detecting that 61% of US patients had their onset of BD prior to the age of 19 years compared to only 30% of European patients. The authors hypothesised an artefact of recruitment bias, healthcare availability and variations in interpretation of the same questions by patient or interviewer, amongst others, as potential reasons for this difference. When patient organisations were surveyed in Europe (Morselli et al. 2003), 33% patients with BD reported an onset before age 20 years.

Another interesting dimension is that of phenomenology of BD in children and adolescents. Geller (Geller and Luby 1997) reported that PBD presents as non-episodic and rapid cycling. Other authors (Leibenluft et al. 2003) have commented that DSM-IV requirements are excessively stringent and have proposed that 1–3 day episodes in juvenile mania as being acceptable for diagnosis. These authors also argue for permitting substitution of grandiosity for elation, and others (Akiskal et al. 2003) have proposed less stringent criteria for psychomotor acceleration. The concept of a rather ‘broad BD phenotype’ was proposed in 2000 (National Institute of Mental Health 2000) and from these discussions, other diagnostic categories such as severe mood dysregulation (SMD) and later disruptive mood dysregulation disorder (DMDD) have been proposed, the latter being subsequently included in DSM 5 (American Psychological Association 2013).

Given the evolving discussion on the nosology of BD, we sought to extend the data presented in Holtmann et al.’s (2010) paper on the rates of diagnosis of BD in children and adolescents who were discharged from inpatient settings in Germany. Here we aim to analyse the national trends in Germany of inpatient visits for the years 2000–2013 with a particular focus on the years 2008–2013, and which significantly exceeds the period covered by Holtmann et al. (2010). We then discuss these trends comparing them to trends for other diagnoses in this age group, and also discuss a possible ‘diagnostic spill-over’ (bringing forward BD diagnosis from adults to younger age groups) by analysing diagnostic categories in the 20–30 year age group. We explored the dataset in an attempt to answer the following questions: (1) What happened to the rates of discharge diagnoses for BD in the period 2008–2013 as well as in the period 2000–2013? (2) Is any change in the rate of discharge diagnoses for BD accompanied by changes in other psychiatric diagnoses during these periods (i.e. is there a diagnostic shift)? (3) Do the rates of discharge diagnoses for BD in adults also vary and is there any evidence of a ‘diagnostic spill-over’ between age groups? These questions are similar as asked in the paper by Holtmann et al. (2010), but we ask these questions of a significantly expanded data set and in the context of diagnostic categories such as SMD and DMDD being discussed in the literature as part of a broader ‘paediatric bipolar phenotype’. In this context, however, we clarify that these diagnostic categories (SMD, DMDD, etc.) do not form part of this dataset, and as such, are not included in our analyses as separate diagnostic categories.

## Methods

We examined the annual reports provided by the German Federal Monitoring System (Bohm and Taubmann 2004) from 2008 to 2013. This is an annual compilation of inpatient discharge diagnoses, using ICD-10 (World Health Organization 1992) criteria, from every hospital (excluding forensic/police hospitals) in Germany. The data are pooled at the national level and are publicly available online (https://www.destatis.de/GPStatistik/receive/DESerie_serie_00000344?list).

The methodology is identical to that of Holtmann et al. (2010). We examined diagnostic discharge rates for BD between 2008 and 2013. We report rates per 100,000 for each age group for the following disorders: (1) BD—Here we report rates of discharge diagnoses for children <15 years old, adolescents 15–19 years and the overall rates in minors 0–19 years old. We also report rates for young adults 20–30 years and for adults above 30 years old. (2) For other psychiatric disorders that maybe related to BD, we report rates per 100,000 for minors 0–19 years old. These disorders are the same as the ones reported by Holtmann et al. (2010) and include substance-related disorders (F10–F19), psychotic disorders (F20–F29), depressive disorders (F32), neurotic, stress-related and somatoform disorders (F40–F48), specific personality disorders (F60), hyperkinetic disorders (F90), conduct disorders (F92), mixed disorders of conduct and emotions (F92) and all mental disorders (F00–F99). The rates per 100,000 were calculated based on population estimates obtained from the Federal Statistical Office (https://www.destatis.de/EN/FactsFigures/SocietyState/Population/Population.html). In the year 2008, the population for minors (0–19 years) was 15,618,736 declining to 14,636,440 by 2013.

Comparisons for 2008 vs 2013 and 2000 vs 2013 were made using odds ratios and their 95% confidence intervals. Statistically significant findings were indicated if the 95% confidence interval did not include the value 1.00. For each discharge psychiatric diagnosis, we computed the proportion of each discharge psychiatric diagnosis as a proportion of all psychiatric diagnoses on discharge in this time period. We mapped long-term trends (2000–2013) in the discharge rates of BD in different age groups, other psychiatric diagnoses in minors as well as proportions of specific psychiatric diagnoses in minors during this overall 14-year period. The level of statistical significance was set and kept at *p* < 0.05. All data were analysed using Stata version 12.1 (College Station, TX: StataCorp LP).

## Results

From 2008 to 2013, there was an 18.3% increase in psychiatric hospital admissions in children and adolescents aged up to 19 years (from 750 to 887 per 100,000), and a 6.5% increase in the mean length of stay (from 18.5 to 19.7 days) resulting in a 17.9% increase in the number of bed-days from about 2.17 million to about 2.56 million. Population figures and the absolute number of inpatient admissions for BD in individuals aged 19 years and under are shown in Table 1.

**Table 1:** Population figures and diagnosis at discharge (absolute numbers) for ICD-10 defined bipolar disorder for individuals 19 years of age and younger in Germany from 2008 to 2013

	2008	2009	2010	2011	2012	2013
Population (million): 0–19 years	15.619	15.339	15.081	14.801	14.660	14.636
BD diagnosed at discharge (years)						
<15	22	26	29	26	27	32
15–19	294	282	314	311	343	328
0–19	316	308	343	337	370	360

**Fig. 1:**
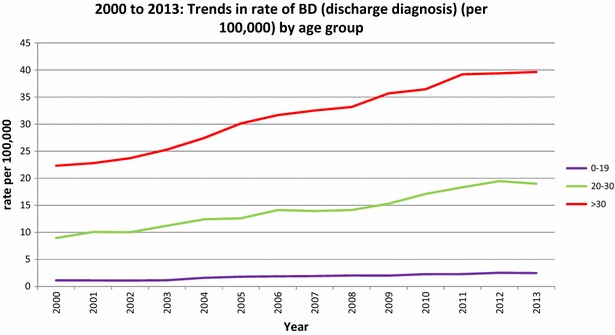
Trends in the rates (per 100,000) of BD as a discharge diagnosis, by age group, from 2000 to 2013

In minors less than 15 years of age, the rate of discharge diagnoses for BD showed a non-significant increase from 0.2 to 0.3 per 100,000 (increase of 50%). In the older age groups, the increase was 24.1% in the 15–19 year olds (from 6.56 to 8.14 per 100,000), 34.4% in the 20–30 year olds (from 14.12 to 18.98 per 100,000) and 19.5% in those over 30 (from 33.17 to 39.63 per 100,000). None of these were statistically significant (Fig. 1).

The increase in hospitalisations for all mental disorders in children and adolescents was linear, but the increase in hospitalisations for BD was fluctuant in absolute numbers but increasing when calculated per 100,000. Notably, the absolute population of children and adolescents showed a linear decline by 6.3% between 2008 and 2013. Also, of note, in 2012, one child under the age of 1 year was recorded as having a diagnosis of BD.

During this interval, increases in admissions per 100,000 individuals up to 19 years were observed for the following ICD-10 discharge diagnoses (in descending order): depressive disorders (F32; 133.6%); neurotic, stress-related and somatoform disorders (F40–48; 22.3%); mixed disorders of conduct and emotions (F92; 8.7%); psychotic disorders (F20–29; 6.5%) and substance-related disorders (F10–19; 1.6%). Decreases in admissions per 100,000 for the same age group were observed for the following diagnoses (in descending order of decline): hyperkinetic disorders (F90; 8.1%), conduct disorders (F91; 2.1%) and personality disorders (F60; 0.6%) (Table 2).

**Table 2:** National rates per 100,000 [odds ratio (OR) and 95% confidence intervals (CI)] for selected ICD-10 defined disorders as discharge diagnoses and age groups in Germany between 2008 and 2013, and between 2000 and 2013

	Age	2000 (1)	2008	2009	2010	2011	2012	2013	OR (95% CI) 2013 vs 2008	OR (95% CI) 2013 vs 2000
Bipolar disorder (F31)	<15	0.14	0.2	0.23	0.27	0.24	0.25	0.3	1.53 (0.89–2.61)	2.03 (1.59–3.55)*
	15–19	3.85	6.56	6.53	7.58	7.72	8.55	8.14	1.24 (1.06–1.45)*	2.11 (1.76–2.53)**
	0–19	1.13	2.02	2	2.27	2.28	2.52	2.46	1.22 (1.05–1.41)*	2.17 (1.83–2.58)**
	20–30	8.95	14.12	15.3	17.09	18.33	19.46	18.98	1.34 (1.26–1.44)**	2.12 (1.96–2.29)**
	>30	22.31	33.17	35.68	36.44	39.2	39.38	39.63	1.19 (1.17–1.22)**	1.78 (1.74–1.82)**
Substance-related disorders (F10–19)	0–19	133.44	220.52	225.82	226.87	236.92	243.94	224.02	1.02 (1.00–1.03)*	1.68 (1.65–1.71)**
Psychotic disorders (F20–29)	0–19	26.02	21.12	20.67	20.99	21.36	21.87	22.49	1.07 (1.01–1.12)*	0.86 (0.83–0.90)**
Depressive disorders (F32)	0–19	12.55	44.68	50.05	65.57	75.31	86.38	104.39	2.34 (2.28–2.41)**	8.34 (7.97–8.72)**
Neurotic, stress-related, somatoform disorders (F40–48)	0–19	98.19	143.6	146.75	159.25	163.24	172.81	175.58	1.22 (1.20–1.25)**	14.15 (13.55–14.79)**
Personality disorders (F60)	0–19	20.75	28.68	29.47	28.7	26.87	28.67	28.5	0.99 (0.95–1.04)	1.37 (1.31–1.44)**
Hyperkinetic disorders (F90)	0–19	20.97	46.86	47.13	45.92	46.76	47.97	44.89	0.98 (0.95–1.01)	2.30 (2.21–2.40)**
Conduct disorders (F91)	0–19	23.20	27.1	28.03	28.82	29.04	28.79	26.52	0.98 (0.94–1.02)	1.14 (1.09–1.19)**
Mixed disorder of conduct and emotions (F92)	0–19	36.75	58.33	61.68	62.9	65.12	66.49	63.4	1.09 (1.06–1.12)**	1.73 (1.67–1.78)**
All mental disorders (F00–99)	0–19	511	750	771	814	847	886	887	1.18 (1.18–1.19)**	1.74 (1.73–1.76)**

* *p* < 0.05

** *p* < 0.001

**Fig. 2:**
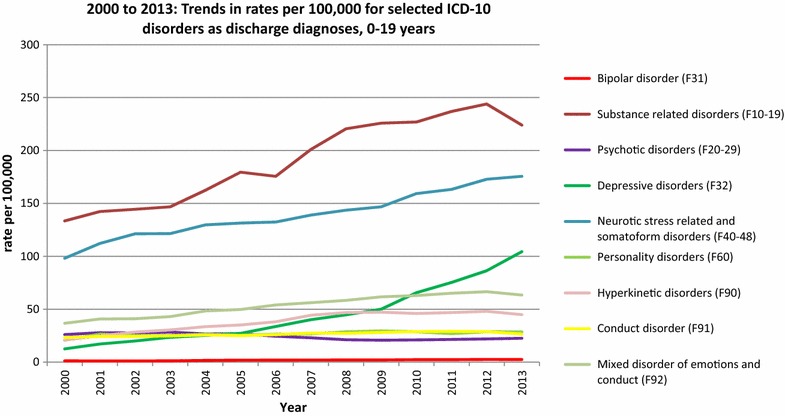
Trends in the rates (per 100,000) for selected ICD-10 disorders as discharge diagnoses

We then compared data from 2000 (when the database was first established) with data from 2013. There was a significant increase in the rate of discharge diagnoses of BD in children less than 15 years of age (0.14–0.3, an increase of over 214%). In the older age groups, there were significant increases across all age groups in the rates of BD as a discharge diagnosis in this period (15–19 years increased by 211%, 20–30 years increased by 211% and >30 years increased by 178%) (Fig. 2).

The figure shows the trends in the rates (per 100,000) for selected ICD-10 disorders as discharge diagnoses in children and adolescents (0–19 years) from 2000 to 2013.

There was an increase in hospitalisation for all categories of mental disorders, with the exception of psychotic disorders, per 100,000, in the period between 2000 and 2013 in the 0–19 age group. In descending order of percentage increase, these were depressive disorders (832%), hyperkinetic disorders (214%), neurotic, stress-related and somatoform disorders (179%), mixed disorders of conduct and emotions (173%), substance-related disorders (168%), personality disorders (137%) and conduct disorders (114%). Interestingly, the rate for psychotic disorders, as a discharge diagnosis, declined by 14% over this period.

The proportion of BD-related discharges in relation to all discharges with mental disorder in children and adolescents up to 19 years increased slightly from 0.26% in 2008 to 0.27% in 2013. Incidentally, this proportion (0.27%) was the same as that in 2007. The proportions of discharges for substance-related disorders, psychotic disorders, personality disorders, hyperkinetic disorders, conduct disorders, and mixed disorders of conduct and emotions in relation to discharges for all mental disorders decreased over the observed period. The proportion of discharge diagnosis of depression increased (5.95–11.78%) as did, marginally, that of neurotic disorders (19.14–19.79%). The proportion of other psychiatric diagnoses marginally increased from 21 to 21.88%.

Interestingly, although the rate of discharge for children with BD less than 19 years old increased significantly between 2000 and 2013, the proportion of BD-related discharges in relation to all mental disorders increased from 0.22 to 0.27.

**Fig. 3:**
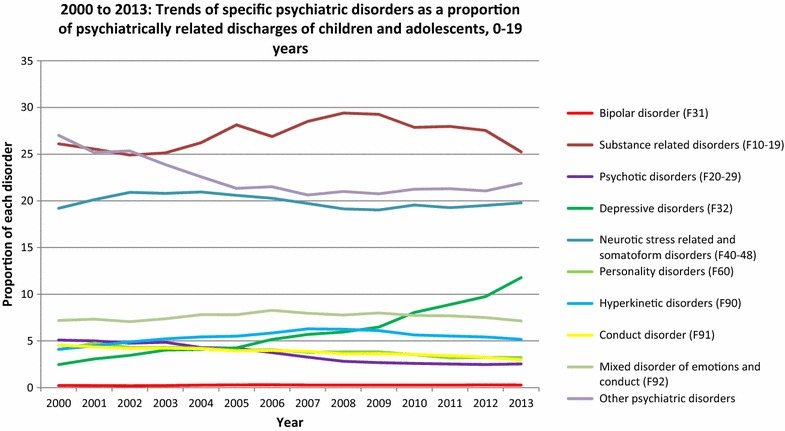
Trends in specific psychiatric disorders expressed as a proportion of all psychiatrically related discharges in children and adolescents from 2000 to 2013

In adults, BD accounted for 2.19% of all psychiatrically related discharges in 2013; the most frequent psychiatric diagnoses were substance-related disorders (37.26%), psychotic disorders (12.14%), neurotic, stress-related disorders (12.14%) and depressive disorders (10.21%). Rates of specific psychiatric disorders as a proportion of the total psychiatrically related discharges are summarised in Table 3 (Fig. 3).

**Table 3:** Specific psychiatric disorders (ICD-10) as a proportion (%) of the total of psychiatrically related discharges of children and adolescents (less than 19 years of age) between 2008 and 2013, and adults in the year 2013 in Germany

	Children and adolescents						Adults
	2008	2009	2010	2011	2012	2013	2013
Bipolar disorder (F31)	0.26	0.26	0.27	0.26	0.28	0.27	2.19
Substance-related disorders (F10–19)	29.4	29.27	27.87	27.97	27.54	25.25	37.26
Psychotic disorders (F20–29)	2.81	2.67	2.58	2.52	2.46	2.53	12.14
Depressive disorders (F32)	5.95	6.48	8.05	8.89	9.75	11.78	10.21
Neurotic, stress-related and somatoform disorders (F40–48)	19.14	19.02	19.56	19.27	19.51	19.79	12.14
Personality disorders (F60)	3.82	3.82	3.52	3.17	3.23	3.21	2.47
Hyperkinetic disorders (F90)	6.24	6.1	5.64	5.52	5.41	5.17	0.05
Conduct disorders (F91)	3.61	3.63	3.54	3.42	3.25	2.98	0.01
Conduct and emotional disorder, combined (F92)	7.77	7.99	7.72	7.68	7.5	7.14	0.01
Other psychiatric disorders	21	20.76	21.25	21.3	21.07	21.88	23.52
All mental disorders (F00–99)	100	100	100	100	100	100	100

## Discussion

Between the years 2008 and 2013, there was an increase in the rates of discharge diagnosis for BD (22%) in the 0–19 age group, which exceeded the general trend of rise in discharges for other mental disorders (18.2%). Holtmann et al. (2010) found a similar trend between 2000 and 2007. We found that the increased rate occurred both in children (<15 years) and in adolescents (15–19 years), although the rise was statistically non-significant in children. The overall trend from 2000 to 2013, however, shows a significant increase in rates of discharge in both children and adolescents for BD. A recent Danish study (Kessing et al. 2014) has also reported an increase (doubling of the rate) in diagnosis of BD in the <19 year age group. This study evaluated nationwide registers of all inpatient as well as outpatient contacts at psychiatric hospitals. The median age at index diagnosis was around 17 years (range 16–18 years) suggesting that the diagnosis was more likely in adolescents.

A recent meta-analysis (Van Meter et al. 2011) of twelve studies (6 US, 6 non-US) showed that the overall rate of BD was 1.8% (range 1.1–3.0%), and that there were no significant differences in the mean rates between US and non-US studies. Here we report a significantly lower (but increasing) rate (2.46 per 100,000 in 2013) of discharges from hospital but we have not included outpatient settings as these data were not available in our dataset. Another significant difference is the inclusion of BD subtypes in some of these studies, whereas our data only included Bipolar Type I (F31, ICD-10). Our results show that the overall rate of discharge with BD as a diagnosis is rather low in Germany but shows an increasing trend over a 14-year period.

The rate of admissions for BD in the older age groups also showed significant increases (20–30 years increased by 34% and >30 years increased by 19%). This increase was noted in both the 2008–2013 period as well as the overall 2000–2013 period. Therefore, similar to Holtmann et al. (2010), we have not found evidence of a diagnostic ‘spill-over’, although we have the same limitation of lacking patient identifiers, thereby limiting longitudinal follow-up that would be necessary to distinguish between new onset BD in young adults versus continuation of a disorder beginning in childhood or adolescence. Table 2 shows that rates for most other disorders increased significantly except for personality disorders, conduct disorders and hyperkinetic disorders, which remained relatively stable.

Interestingly, when analysing the long-term trends (2000–2013), the rates of admission for all other mental disorders increased significantly except for the rates of psychotic disorders, which fell by almost 14% in this period. One possible reason could be a reverse diagnostic ‘spill-over’ where minors previously diagnosed with psychotic disorders were being given a diagnosis of BD. Notably, BD as a proportion of all psychiatrically related discharges increased from 0.22 to 0.27% in this period. Equally however, psychotic disorders could also have been replaced by other disorders such as substance-related disorders which have also shown a significant increase in this period and therefore, from this dataset, one cannot reasonably infer the reverse diagnostic spill-over phenomenon. Another possibility could be an increase in the quality and number of community-based early intervention treatments for psychotic disorders in recent times. However, at this stage, such assumptions remain speculative, and further research on the long-term trends for rates of psychotic disorders and aetiological factors is needed, which is beyond the scope of this paper.

A diagnostic “up-coding” to counteract assumed changes of the German insurance companies’ reimbursement policies seems an unlikely explanation for the observed changes, since one of the most severe diagnostic categories, i.e. psychotic disorders, was diagnosed less often. It is important to note that based on the current data, it is not possible to conclude that there actually was an increase in the prevalence of BD as a diagnosis in Germany in the index period.

It may be hypothesised that the increase in hospitalisation rates for mental disorders from 2008 to 2013 is related to rising inpatient capacity in terms of available beds in the same time period. Of note, there was a steep reduction in available inpatient treatment capacity (=available beds) for child and adolescent psychiatry in Germany from the 1990s (8.316 beds per 100,000 population in 1991 to 5.7 beds per 100,000 population in 2003 and further declining) (Salize et al. 2007). Inpatient capacity increased from 5.168 beds per 100,000 in 2008 to 5.941 beds per 100,000 in 2013 (an increase of 15%). A concomitant slight increase occurred in adult inpatient beds (from 53.061 to 54.433 beds per 100,000 population, an increase of 2.6%) (Statistisches Bundesamt 2013). It remains speculative as to how far the rise in inpatient capacity could have resulted in an increased demand for psychiatric treatment. However, the higher availability of beds does not explain the observed differential rise in the different diagnostic categories of severe mental disorders.

A further interesting and noticeable trend is the 730% increase in the diagnosis of depression at discharge in minors. A detailed analysis of potential reasons is beyond the scope of this paper, but different factors related to this phenomenon need to be taken into consideration such as changes in prevalence of depressive disorders in minors, as well as increased awareness of such disorders which may lead families to seek professional help. One review (Hidaka 2012) identified the issue of possible increase in prevalence of depression (across age ranges) and also discussed potential aetiological factors. It is noteworthy that research in this area is not consistent and that a meta-analysis (Costello et al. 2006) in the last decade that included 26 studies from various countries did not find an increased prevalence of child and adolescent depression over 30 years. A potential area for future research could be mapping the trends identified in this paper against sociodemographic factors (and other potential aetiological factors) in Germany over this period. While this study sought to understand trends in the diagnosis of BD in minors, the two examples above highlight some interesting trends in other disorders and a future study could evaluate rates of diagnosis at discharge of other common disorders.

Although the current dataset is unable to test for this hypothesis, the ‘broad phenotype’ of BD highlights an important issue that could be the focus of future research when analysing differences in diagnostic rates of BD. Irritability and anger are symptoms that have been debated for over a decade and there have been varying conclusions from previous research that have been summarised by Leibenluft (2011). Mood instability and irritability (including anger) where young people present with hyperarousal and non-episodic irritability without the classic well-demarcated periods of elevated or irritable mood that is considered characteristic of BD have been sought to be captured under a category known as severe mood dysregulation (SMD). From research on this proposed category came the diagnostic category entitled disruptive mood dysregulation disorder (DMDD), which has now been included in DSM-5 (American Psychological Association 2013) as a distinct disorder under the category of mood disorders. This approach has since been criticised by the World Health Organisation (WHO) task force (Lochman et al. 2015), mainly due to the research on SMD being in its early days, mostly focussed in the USA, and the adaptation to DMDD without adequate peer-reviewed research and limited support for DMDD in field trials. Moreover, concerns have been expressed that DMDD would, in fact, add to the diagnostic confusion rather than alleviate it. Another suggestion (Runions 2015) is separating the constructs of oppositional defiant disorder (ODD) (into irritability and non-compliance/oppositional behaviours) so that the issue of ‘irritability’ is examined across the spectrum of BD, SMD/DMDD and ODD.

While there is growing consensus that chronic non-episodic irritability does not constitute BD (Grimmer 2014), it could be argued that the above constellation of symptoms results in severe impairments that, in the absence of appropriate diagnostic labels, suffer from lack of treatment. Possibly, clinicians use BD, as a diagnosis, to support access for this group of young people to the mental health system. We acknowledge that the current dataset is unable to test for this hypothesis, as the diagnostic categories of DMDD/SMD are not included in these data.

## Limitations

Similar to Holtmann et al. (2010), the dataset consists of administrative rather than clinical data points and it is not possible to separate dual/multiple diagnoses in patients. The reported diagnoses are not made using standardised tools and we are reliant upon the treating physician using the ICD-10 (World Health Organization 1992) in an empirical manner. The data were not originally collected for research purposes and therefore reliability and validity are unknown. As the data obtained are cross-sectional, we have no way of ascertaining any changes of diagnosis in individual clients over time, or indeed, any co-morbidity. We are, therefore, also unable to account for multiple admissions for the same individual as the data are ‘sterile’ with no patient identifiers. The rates calculated are for discharges from inpatient units only and do not include outpatient rates of diagnoses, which is an important limitation.

However, we have been able to compare our data with Holtmann et al. (2010) using a like dataset and have been able to evaluate long-term (14 years) trends in inpatient admission diagnostic rates. This, along with the availability of data publicly, allows for verification of the results and can be considered as strengths of the study. It is also noteworthy that the dataset is complete with no identified missing values, which again is a particular strength of the study.

## Conclusions

The rate of BD as a discharge diagnosis in children and adolescents in Germany has increased significantly over a 14-year period, consistently exceeding the general trend for a rise in discharges for mental disorders over a period of 14 years. However, the rate of rise is modest and BD is still only a small proportion of all mental disorder diagnoses in child and adolescent psychiatric inpatients in Germany. We have also found no clear evidence for a ‘diagnostic shift’ between other psychiatric diagnostic categories or a ‘diagnostic spill-over’ between age groups. We have not directly compared BD diagnoses across countries/regions but the strengths and limitations of our study suggest the need for comparative epidemiological studies across world regions using formal diagnostic criteria. Empirical diagnostic criteria for the various phenotypes of bipolar disorder are necessary for epidemiological studies to have relevance ‘at the coal face’ and efforts must continue to refine the various subtypes of BD with ongoing field trials. Finally, although this study focussed on BD, the dataset shows a highly significant rise in the rate of discharge diagnosis for depressive disorder as well as a significant decline in rates for psychotic disorders in children and adolescents in Germany. This is in variance with research from other sources and ought to be investigated.
